# The Using of Concrete Wash Water from Ready Mixed Concrete Plants in Cement Systems

**DOI:** 10.3390/ma14102483

**Published:** 2021-05-11

**Authors:** Danutė Vaičiukynienė, Aras Kantautas, Simona Tučkutė, Fallon Manhanga, Eugenijus Janavičius, Ernestas Ivanauskas, Žymantas Rudžionis, Aloyzas Gaudutis

**Affiliations:** 1 Faculty of Civil Engineering and Architecture, Kaunas University of Technology, Studentu St. 48, LT-51367 Kaunas, Lithuania; aras.kantautas@ktu.lt (A.K.); fallon.manhanga@stud.ktu.edu (F.M.); ernestas.ivanauskas@ktu.lt (E.I.); zymantas.rudzionis@ktu.lt (Ž.R.); 2 Center for Hydrogen Energy Technologies, Lithuanian Energy Institute, Breslaujos St. 3, LT-44403 Kaunas, Lithuania; simona.tuckute@lei.lt; 3 JSC “Concretus Grupė”, Vytenio St. 4, LT-03113 Vilnius, Lithuania; e.janavicius@concretus.lt; 4 Faculty of Water and Land Management Agriculture Academy, Vytautas Magnus University, Studentu St. 11, LT-53361 Akademija, Lithuania; aloyzas.gaudutis@gmail.com

**Keywords:** concrete wash water, zeolitic by-product, hydration temperature

## Abstract

Concrete plants accumulate large amounts of concrete wash water. This water, which pH is highly alkaline, has a negative impact on the environment. Its reuse in fresh concrete slightly reduces its mechanical properties. The combination of concrete wash water and zeolitic by-product led to an increase of 4.6% in the compressive strength at 7 days hydration and up to 30% at 28 days hydration. The same combination led to the denser microstructure compared to the samples made with concrete wash water. This could be explained by the pozzolanic reaction of the zeolitic by-product. The complex chemical reactions of cement, zeolitic by-product, and fines presented in the concrete wash water occurred. Therefore, it was suggested the reusing method of concrete wash water together with zeolitic by-product in the fresh concrete mixtures by substituting some amount of tap water with concrete wash water. In this way, the consumption of tap water is possible to reduce in cement systems.

## 1. Introduction

In ready-mixed concrete plants, the recycling of wash wastewater is an actual problem. The wash wastewater is accumulated from the washing of concrete mixing trucks and batching plants. In Lithuanian concrete plants, this wastewater is generated in the settling ponds and it could be led to problems of environmental impact. Concrete wash water has high pH values, and it could pollute local water sources or soils [1]. However, the system can be restricted by limited space, residential expansion, weather, and other factors.

One of the environmentally friendly solutions could be the recycling of concrete wash water in the preparation of fresh concrete. Asadollahfardi et al. [2] stated that concrete wash water did not significantly change the compressive strength of concrete but decreased the setting times. Similar results were obtained by Sandrolini et al. [3]. The samples of mortar and concrete made with concrete wash water had slightly lower compression strength than the samples which was made with tap water. The use of concrete wash water led to a decrease in the water absorption which is closely related to the durability of the samples. Chatveera et al. [4,5] used the concrete wash water in the mixing water for the preparation of concrete samples. It was concluded that by increasing the amount of concrete wash water in fresh concrete the compressive strength and the slump decreased. Similar results were obtained for the concrete samples with fly ash as an additive or a superplasticizer admixture. Ružinski et al. [6] stated that concrete wash water did not reduce the compressive strength of concrete samples significantly and this decrease of strength did not exceed 90 percent compared with control samples produced with tap water. Concrete wash water has new possibilities for recycling in fresh concrete mixtures, but specific corrections in the mix should be done as reported by Ferriz-Papi et al. [7]. de Matos et al. [8] identified that concrete wash water had a positive impact on the development of compressive strength of concrete samples. After 3 and 7 days of hydration, the compressive strength increased 8 and 16%, respectively, by comparing with the control samples where tap water was used. At the age of 28 days, the compressive strength of the samples was detected slightly lower, and it consisted 92% of the strength for reference sample. The concrete wash water did not change the significant main properties of fresh and hardened mortar as stated by Aboelkheir et al. [9]. In this case, the strength reached above 90% of mortar samples with concrete wash water compared with the samples which were made by using tap water.

Therefore, the incorporation of concrete wash water in the production of mortar or concrete is related to the decreased strength values in many of the cases studied. Many researchers [10,11,12,13] have studied the incorporation of zeolites in cement systems. When in the cement systems zeolite was introduced, SiO_2_ and Al_2_O_3_ in zeolite reacted with Ca(OH)_2_ which formed during cement hydration. During the pozzolanic reaction, the binding phases such as calcium of silicate hydrate and calcium aluminate hydrate formed. These hydrates have a positive effect on the mechanical properties of cement systems. Markiv et al. [10] concluded that concrete containing clinoptilolite had lower compression strength at 90 days of hydration but the strength became higher after 180 days by comparing with the strength of concrete without zeolite. The substitution of cement by clinoptilolite resulted in the formation of calcium silicate hydrate, hydrogelenite, and ettringite which had a positive effect on the durability of these concretes. Tran et al. [11] also reported that the incorporation of zeolites in the systems of concrete improved durability and mechanical properties. Girskas et al. [12] stated that the substitution of Portland cement with the mixture of NaA and NaX zeolites up to 10% of increased the freeze-thaw resistance of this concrete compared to concrete without zeolite. Najimi et al. [13] also found that the use of 15 wt % clinoptilolite resulted in the increased strength and durability of concrete.

In this paper, the effect of concrete wash water with the additive of zeolitic by-product on the main properties of hardened cement paste was investigated. The impact of zeolitic by-product on the hydration temperature, compressive strength, mineral composition, and microstructure was evaluated. The investigated results were compared between the samples with incorporated zeolitic by-product and without it.

## 2. Materials and Methods

The chemical compositions of Portland cement and zeolitic by-product were investigated by an X-ray fluorescence spectrometer, a Bruker X-ray S8 Tiger WD (Bruker AXS GmbH, Karlsruhe, Germany), using a rhodium (Rh) tube, an anode voltage Ua up to 60 kV, and an electric current I up to 130 mA. Pressed samples were analyzed in a helium atmosphere. Measurements were performed using the SPECTRA Plus QUANT EXPRESS method [14].

The mineral composition of initial materials such as Portland cement and zeolitic by-product and hardened cement paste were evaluated by using the X-ray diffraction analysis (XRD). This analysis of the materials was performed using the X-ray diffractometer D8 Advance diffractometer (Bruker AXS GmbH, Karlsruhe, Germany). CuKα radiation and Ni filter were used. The power X-ray diffraction patterns were identified with references available in PDF-2 database (PDF—2 International Centre for Diffraction Data, 12 Campus Boulevard Newtown Square, PA 19073-3273 USA) [15].

The particle size distribution of the zeolitic by-product was determined by using a laser particle size analyzer (CILAS 1090 LD, Orleans, France). The distribution of solid particles in the air stream was 12–15 wt.%. Compressed air (2500 mbar) was used as the dispersing phase. The measurement time was 15 s [16]. The specific surface was measured with the Blaine instrument according to the EN 196-6 standard [17].

The microstructures of the materials were studied by scanning electron microscope (SEM). A high-resolution scanning electron microscope ZEISS EVO MA10 (Carl Zeiss AG, Oberkochen, Germany) was used for the research [18].

The hydration temperature measurements of Portland cement paste were performed with 8-channel USB TC-08 Thermocouple Data Logger (Pico Technology Limited, Cambridgeshire, UK) (temperature measurements range from −270 to +1820 °C).

The pH measurements of concrete wash water were conducted by using a Hanna ISE pH meter (Hanna Instruments, Limena, Italy). Chlorides were determined according to the method prescribed by LAND 63-2004 [19]; nitrite—LAND 39-2000 [20]; nitrate—LAND 65-2005 [21]; total nitrogen—LAND 59-2003 [22]; phosphate—LAND 58-2003; total phosphorus—LAND 58-2003 [23]; zinc—LST ISO 8288:1998/P:2009 [24]; lead—LST ISO 8288:1986 [25]; mercury—LST EN ISO 12846:2012 [26]; total solids ISO 15587-2:2002 [27].

To determine the compressive strength of the hardened cement paste, 2 × 2 × 2 cm cubes were formed from cement paste of normal consistency. The water/solid material ratio of the mixture (normal consistency) was determined according to the standard EN 196-3 [28]. The compressive strength of the samples was determined with the press ELE AutoTest.

The samples of hardened cement paste were based on Portland cement (OPC) which selected for the CEM I 52.5R type. The chemical composition of OPC and zeolitic by-product was shown in Table 1. Some mount of OPC was substituted with the powder of zeolitic by-product. This zeolitic material (spent fluid catalytic cracking (FCC) catalyst) was generated by the oil industry. The oxides of silicon and aluminum dominated, and the sum of these oxides consisted roughly 84% (Table 1). Zeolitic by-product was polluted with the insignificant amount of oil products [29]. Despite these organic compounds impurities, zeolitic by-product exhibits excellent pozzolanic properties in Portland cement systems [30,31]. The chemical composition of zeolitic by-product should be stable because it is based on synthetical faujasite type zeolite. Thus, the composition depends on the manufacturer which produces this zeolite and, in the process, used in oil plant. The quantity of the zeolitic by-product directly depends on the productivity of the oil industry. In Lithuania, about 200 tons of this by-product is generated per year [32] and it accumulates in landfill.

The surface area of Portland cement (350.0 m^2^/kg) is almost 2.5 times large compared with the surface area of zeolitic by-product (142.1 m^2^/kg).

**Figure 1:**
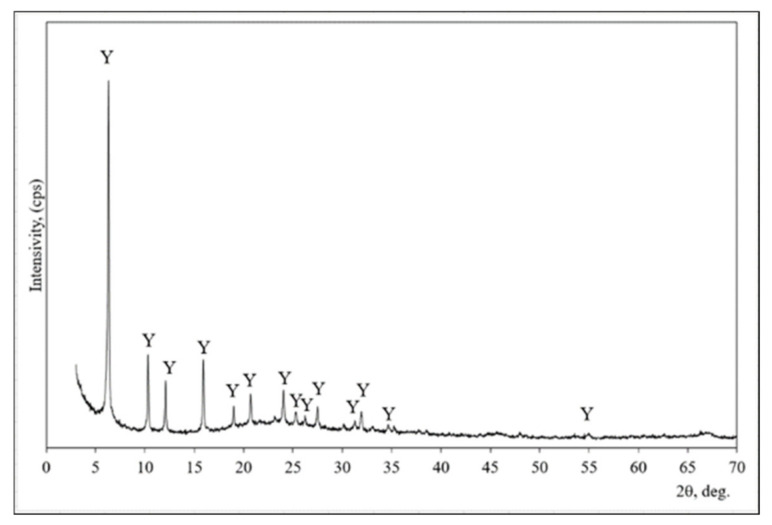
The X-ray diffraction pattern of zeolitic by-product. Note: Y is faujasite (73-2312) H_7.7_ Al_42.56_∙Si_139_∙O_345.6_.

XRD analysis was used for the mineral composition evaluation of zeolitic by-products (Figure 1). This material is composed of faujasite type zeolite [33]. All peaks are assigned to faujasite (interplanar distances (d) of 1.404; 0.859; 7.330; 5.577; 4.679; 4.297; 3.707; 3.249; 2.807 nm) in the X-ray diffraction pattern.

**Figure 2:**
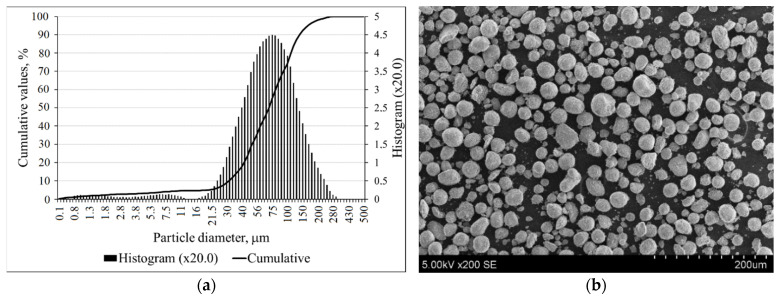
The particle size distributions (**a**); SEM image (**b**) of zeolitic by-product.

The particle size distribution of zeolitic by-product is shown in Figure 2a, and these particles had a mean particle diameter of 78.39 µm. According to SEM, the crystals had a round, spherical shape (Figure 2b).

The concrete wash water was received from the ready-mix truck of a Lithuanian concrete company. In Table 2 the main characteristics of this water were presented.

The high pH value was detected for this concrete wash water and it could be related to the high content of alkalis from Portland cement in the water (Table 2). Without dissolved calcium compounds other solids such as sulfates, chlorides, nitrite, and nitrate were detected as well as reported by Sandrolini et al. [3]. All these chemical compounds could be related to the hydration of OPC and appear in the chemical admixtures of concrete. The concrete wash water contains residual cement particles and the chemical superplasticizer (polyelectrolytes) as water reducer and retardant as well [1]. The heavy metals consisted only of traces in the concrete wash water (Table 2). In addition to the concrete wash water, tap water was used for the dilution.

Two types of hardened cement paste samples were analyzed (Table 3). In the first type, zeolitic by-product was not incorporated. Five series of samples were formed by changing the ratio between concrete wash water (WW) and tap water (TW). In the second type of hardened cement paste, 5 wt.% of zeolitic by-product was inserted. The amount of zeolitic by-product was chosen according to our previous study [34]. The water and solid ratio in all mixtures was the same (W/S = 0.35). First, all dry components such as Portland cement and zeolitic by-product were mixed. Then, the water was filled, and the pastes were mixed again to a homogenous mass for 4–5 min.

## 3. Results and Discussion

**Figure 3:**
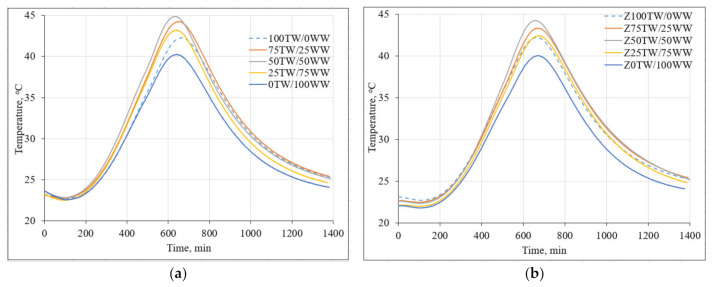
The hydration temperature of hardened cement pastes: (**a**) with concrete wash water; (**b**) with the combination of concrete wash water and zeolitic by-product.

Figure 3 demonstrates the impact of concrete wash water on the hydration temperature of hardened cement pastes. The measurement results showed that the high alkalinity of concrete wash water led to the decrease of setting time of cement pastes (Figure 3a). The main peak of 100TW/0WW sample reached the maximal temperature at 688 min. Meanwhile, the main peaks times of samples with concrete wash water such as 75TW/25WW, 50TW/50WW, 25TW/75WW, 0TW/100WW were slightly shorter: 671 min, 645 min, 650 min and 651 min, respectively compared with the time of 100TW/0WW sample. Similar results were published by Asadollahfardi et al. [2]. They determined that concrete wash water decreased the setting time of OPC. The additional amount of calcium ions from concrete wash water correlated with the faster precipitation of calcium hydroxide (CH) and C-S-H and the acceleration of the cement hydration [35].

In the second type of hardened cement paste samples (with 5 wt.% of zeolitic by-product) the main peaks reached almost the same time (Figure 3b). The incorporation of zeolitic by-product led to the binding of Ca(OH)_2_ which formed during the hydration of Portland cement and from concrete wash water as well. In this case, cement-like hydrated products are generated during the hydration process [36].

The lowest hydration temperature of the cement pastes 40.2 °C and 40.0 °C was detected for 0TW/100WW and Z0TW/100WW samples respectively. This could be explained by the high content of dissolved solids, compared to the tap water. Due to the common ion effect, the rate of cement hydration slowed down [9]. The higher hydration temperatures were for the pastes produced with the water mixtures of tap water and concrete wash water. In both types, the highest hydration temperatures were reached for the cement pastes where 50 wt.% of tap water was substituted with 50 wt.% concrete wash water. It was determined that the substitution of tap water with concrete wash water had an insignificant influence on the maximal hydration temperature in the samples of cement pastes [4].

**Figure 4:**
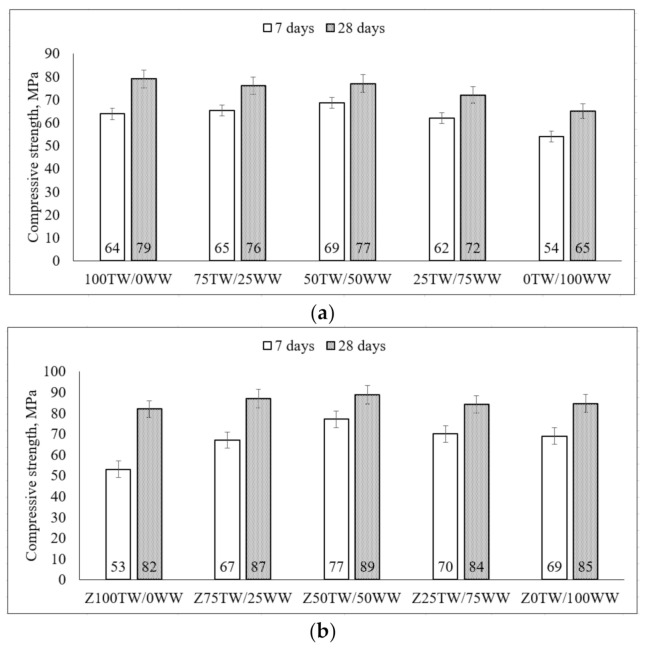
The compressive strength of hardened cement pastes with concrete wash water (**a**) and with the combination of concrete wash water and zeolitic by-product (**b**) at 7 days and 28 days.

Li et al. stated that [37] the good linear correlation of hydration exothermic peak and compressive strength with the correlation coefficient was 0.8–1 was determined. At 7 days, the concrete wash water slightly reduced the strength up to 84% compared with the compressive strength of 100TW/0WW samples (Figure 4a). For some compositions such as 75TW/25WW, 50TW/50WW, it was slightly higher than the compressive strength of 100TW/0WW. After 28 days of hydration, the slightly lower compressive strength had the samples with concrete wash water compared to the samples without concrete wash water (Figure 4a). Similar results of compressive strength were published by Chatveera et al. [4]. They determined that the hardened cement paste had a more porous and weaker matrix due to the high alkalinity environment and total solid content of concrete wash water. Therefore, at 7 days of hydration, the concrete wash water led to a slight increase of compressive strength but after longer hydration time (at 28 days) the strength was reduced up to 82% compared with 100TW/0WW samples. Similar tendencies of strength were detected by de Matos et al. [8] and Ekolu et al. [38].

When in the system zeolitic by-product was incorporated then both compressive strength (at 7 and 28 days) increased by increasing the amount of concrete wash water (Figure 4b). In this case, by using the combination of zeolitic by-product and concrete wash water the values of compressive strength were higher than the strength of samples prepared using only concrete wash water. At the early ages, the zeolitic by-product reacted with Ca(OH)_2_ from concrete wash water leading to a rise in compressive strength [5,6]. Later the compressive strength (at 28 days) was improved due to the pozzolanic reaction of zeolitic by-product [39]. Chatveera et al. [5] got the higher compressive strength of concretes made with concrete wash water and fly ash compared to the strength of concrete with tap water. The compressive strength of samples containing 50 wt.% tap water plus 50 wt.% concrete wash water (Z50TW/50WW) indicated relatively more strength up to 89 MPa [2].

**Figure 5:**
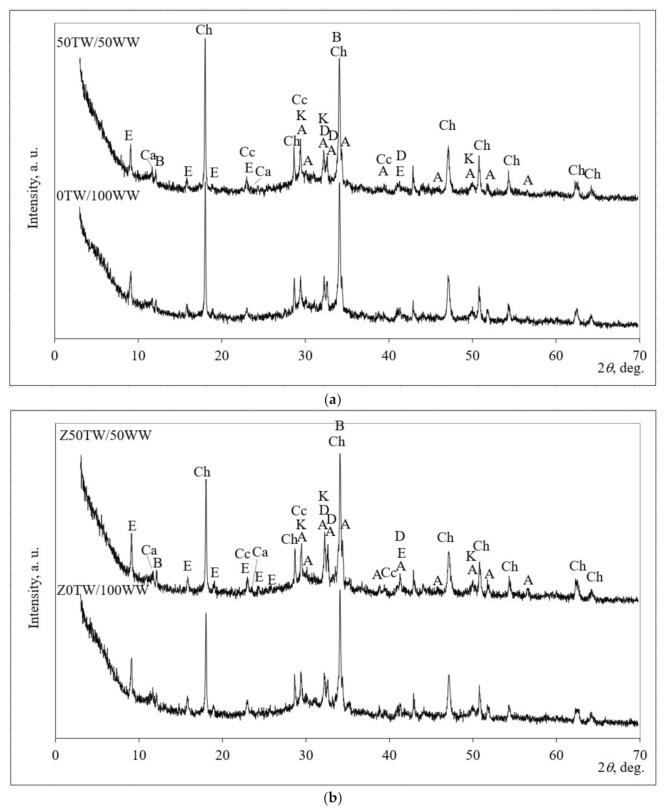
X-ray diffraction (XRD) pattern of hardened cement paste: (**a**) with concrete wash water; (**b**) with the combination of concrete wash water with zeolitic by-product at 28 days of hydration. Notes: Ch—portlandite Ca(OH)_2_ (84-1265); CC—calcite CaCO_3_ (5-586); E—ettringite Ca_6_Al_2_(SO_4_)_3_(OH)_12_·26H_2_O (41–1451); A—alite Ca_54_MgAl_2_Si_16_O_90_ (13-272); D—belite Ca_2_(SiO_4_) (83-461); K—calcium silicate hydrate Ca_1.5_Si O_3.5_∙*x*H_2_O (33-306); B—brownmillerite Ca_2_(Al,Fe)_2_O_5_ (30-226), Ca—calcium aluminum oxide carbonate hydrate Ca_4_Al_2_CO_9_ ∙11H_2_O (14-83).

The mineral composition was determined by using XRD patterns (Figure 5). The mineral compositions of four samples (0TW/100WW, 50TW/50WW, Z0TW/100WW and Z50TW/50WW) were analyzed. These samples were chosen due to the highest compressive strength of the above-mentioned compositions (Figure 4).

Similar mineral composition of hardened cement paste was detected for samples with concrete wash water and for the samples with the combination of zeolitic by-product and concrete wash water as well. Tran et al. [40] determined that concrete wash water did not have a significant impact on the hydration chemistry of ordinary Portland cement (OPC). In all hydrated samples the peaks of portlandite (Ch), calcium silicate hydrate (K), calcite (CC), ettringite (E), and calcium aluminum oxide carbonate hydrate (Ca) were detected in the hardened cement paste after hydration of 28 days. Alite (A); larnite (D), and brownmillerite (B) were detected as well as anhydrate minerals of Portland cement. However, the XRD patterns of all hardened cement pastes demonstrated different intensities of the main peaks of portlandite and ettringite. When in the system zeolitic by-product was incorporated (Figure 5b), the slightly lower main peak of portlandite at about 18.0° 2*θ* and slightly higher peaks of ettringite at about 9.1°, 15.9°, and 19.1° 2*θ* were determined compared with the samples without zeolitic by-product (Figure 5a). The peaks related to zeolitic by-products (faujasite) were not detected. This could be explained by the pozzolanic reaction of the zeolitic by-product. During the hydration process, the aluminosilicate network of zeolitic by-product decomposed and calcium cations reacted with aluminate and silicate radicals by forming calcium silicate hydrate and calcium aluminum oxide carbonate hydrate [41].

**Figure 6:**
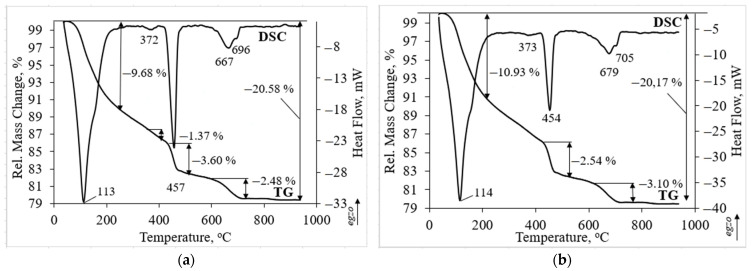
Thermal analysis of hardened cement paste samples after 28 days: (**a**) 50TW/50WW sample; (**b**) Z50TW/50WW sample.

Thermal analysis has confirmed the findings of XRD analysis. Thermal analysis was performed on the sample with the highest compressive strength (Figure 6). In both investigated samples three main peaks could be detected. The first endothermic peak at about 113 and 114 °C temperatures is related to the dehydration of hydrated products such as calcium silicate hydrate and ettringite [8]. The ettringite peaks do not clearly visible due to overlapping with the calcium silicate hydrate peak [42]. The second endothermic peak at about 457 and 454 °C temperature is attributed to the decomposition of portlandite. The third peak was detected as a double peak. The endothermic peak at 667 and 679 °C temperature is assigned to the water removal from the calcium silicate hydrate and to the decarbonation of carbonated phases as well [43]. Decarbonation of calcite is related to the peak at higher temperature of 696 and 705 °C [44]. In addition to the main and intensive peaks the endothermic peak at about 372 and 373 °C temperature is related to the dehydration of hydrated aluminates such as calcium aluminum oxide carbonate hydrate which was detected in XRD patterns as well (Figure 5) [30].

The incorporation of zeolitic by-product changed the intensity of the main endothermic peaks. Due to pozzolanic reactions of zeolitic by-product (Z50TW/50WW sample), the peak related to portlandite decomposition was weaker compared to the 50TW/50WW sample (Figure 6b). In this case, the mass loss consisted slightly lower value of 2.54 wt.% compared with the sample without zeolitic by-product (3.60 wt.%). Rahhal et al. [39] stated that the crystals of zeolitic by-product decomposed and reacted with Ca(OH)_2_ after incorporation in the cement paste. It is possible that the larger amount of hydrated products such as calcium silicate hydrate and ettringite was formed. This statement is confirmed by the mass loss at temperatures up to 225 °C. It was detected a slightly higher mass loss of 10.93 wt.% for the Z50TW/50WW sample compared to the sample without zeolitic by-product where the mass loss was 9.68 wt.% (Figure 6a). These findings were confirmed XRD analysis. By using the mixture of concrete wash water and zeolitic by-product it was reached a slightly higher degree of OPC reaction [8].

**Figure 7:**
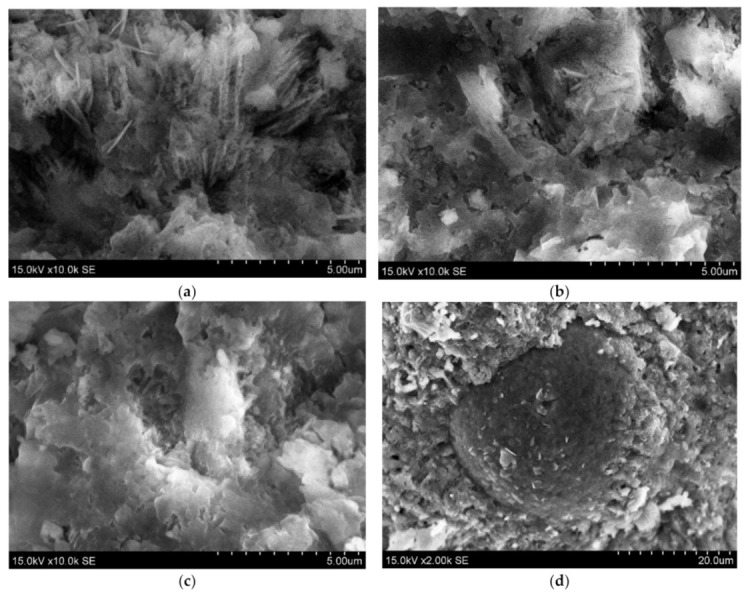
The morphologies of hardened cement paste at 28 days of hydration: (**a**,**b**) 50TW/50WW sample; (**c**,**d**) Z50TW/50WW sample.

The analysis of scanning electron microscopy (SEM) was performed for the evaluation of microstructure (Figure 7). It was detected a relatively porous matrix of Z50TW/50WW sample (Figure 7a,b). Chatveera et al. [4] stated that the incorporation of concrete wash water in the cement systems led to the formation of a more porous and weaker matrix compared to the matrix of samples with tap water. The mixture of concrete wash water and zeolitic by-product (Z50TW/50WW) had an influence on the microstructure of the hardened cement paste. It became denser compared with the microstructure of the samples with concrete wash water (Figure 7c). This change of microstructure could be related to complex chemical reactions of OPC, zeolitic by-product and the fines present in the concrete wash water [3,45]. The round particle of zeolitic by-product is coated with hydration products due to the pozzolanic effect is detected in (Figure 7d).

These results indicated that concrete wash water could be an alternative and sustainable source of cement systems. The recycling of concrete wash water together with zeolitic by-products in the fresh concrete mixtures could be considered a very promising sustainability key.

## 4. Conclusions

The high alkalinity of concrete wash water led to the decrease of setting times of cement pastes. Meanwhile, the mixture of concrete wash water and zeolitic by-product almost do not change the times of the main peak.

The compressive strength of samples with the combination of concrete wash water and zeolitic by-product led to an increase of 30% in the compressive strength at 7 days hydration and up to 4.6% at 28 days hydration compared with Z100TW/0WW samples. This increase could be explained by the reaction of zeolitic by-product and Ca(OH)_2_ from the concrete wash water and cement hydration.

It was determined that concrete wash water with zeolitic by-product did not have a significant impact on the hydration chemistry of Portland cement (OPC). It slightly increased ettringite and decreased the amount of portlandite in hardened cement paste. In this case, the mass loss consisted slightly lower value of 2.54 wt.% compared with the sample without zeolitic by-product (3.60 wt.%). This decrease could be explained by the pozzolanic reactions of the zeolitic by-product: it decomposed and reacted with Ca(OH)_2_ and after that, the larger amount of hydrated products such as calcium silicate hydrate and ettringite was formed.

So, these results indicated that concrete wash water could be an alternative and sustainable source of cement systems. The recycling of concrete wash water together with zeolitic by-products in the fresh concrete mixtures could be considered a very promising sustainability key.

## Figures and Tables

**Table 1:** Chemical composition of the zeolitic by-product, wt.%.

Oxides	OPC CEM I 52.5R	Zeolitic by-Product
SiO_2_	21.00	35.40
Al_2_O_3_	3.90	48.77
Fe_2_O_3_	2.90	1.02
La_2_O_3_	n	1.63
TiO_2_	n	3.57
MgO	2.70	0.44
CaO	66.00	0.37
Na_2_O	n	0.312
SO_3_	3.40	0.07
P_2_O_5_	n	0.08
K_2_O	n	n
Cl	0.06	2.57
Other	n	5.77
Bulk Density, kg/m^3^	1236	864
Specific Density, kg/m^3^	3122	2679
Surface Area (Blaine), m^2^/kg	350.0	142.1

**Table 2:** Concrete wash water characteristics.

Parameter	Units	Value
pH		12.37
Chlorides	mg/L	<4.5
Nitrite Ion	mg/L	0.022
Nitrate Ion	mg/L	0.14
Total Nitrogen	mg/L	3.3
Phosphate	mg/L	0.16
Total Phosphorus	mg/L	1.6
Sulphate	mg/L	19
Calcium	mg/L	867
Zinc	mg/L	0.94
Mercury	mg/L	0.33
Lead	mg/L	0.17
Total Solids	mg/L	852

**Table 3:** The quantity of initial materials for the composition of cement pastes.

Samples	Portland Cement (wt.%)	Zeolitic by-Product (wt.%)	Tap Water (wt.%)	Concrete Wash Water (wt.%)	W/S ^1^
100TW/0WW	100	0	100	0	0.35
75TW/25WW	100	0	75	25	0.35
50TW/50WW	100	0	50	50	0.35
25TW/75WW	100	0	25	75	0.35
Z0TW/100WW	100	0	0	100	0.35
Z100TW/0WW	95	5	100	0	0.35
Z75TW/25WW	95	5	75	25	0.35
Z50TW/50WW	95	5	50	50	0.35
Z25TW/75WW	95	5	25	75	0.35
Z0TW/100WW	95	5	0	100	0.35

^1^ The ratio of water and solid materials.

## Data Availability

Not applicable.
